# Supplementary material for: Biological and health-related effects of weak static magnetic fields (≤ 1 mT) in humans and vertebrates: A systematic review
Source: PLoS One. 2020 Jun 9;15(6):e0230038. doi: 10.1371/journal.pone.0230038 (PMC7282627; doi:10.1371/journal.pone.0230038)
Supplement: S1 Table — (DOCX) [file pone.0230038.s002.docx]

**S1 Table.** **Schematic overview for placement of individual human and animal studies in study quality categories (1 st tier, 2nd tier, 3rd tier).** Adapted from the approach recommended by the National Toxicology Program's Office of Health Assessment and Translation (2015)^1^. “++” definitely low risk of bias, “+” probably low risk of bias, “-“ probably high risk of bias, “--“ definitely high risk of bias

|  | | | | **Key criteria** | | | **Other criteria** | | | | | |
| --- | --- | --- | --- | --- | --- | --- | --- | --- | --- | --- | --- | --- |
| **Category** | **Guidance** |  |  | **Identical experimental conditions** | **Confidence exposure characterization** | **Confidence outcome assessment** | Randomization exposure level | Allocation concealment | Blinding | Attrition/Exclusion rate | Reporting | Other potential threats |
| 1st tier | “definitely low” or “probably low” risk of bias for all key criteria  AND  “definitely low” or “probably low” risk of bias for more than half of the other applicable criteria |  |  |  |  |  |  |  |  |  |  |  |
|  |  | Example 1 | | ++ | ++ | + | + | - | - | ++ | + | + |
|  |  |  |  |  |  |  |  |  |  |  |  |  |
| 2nd tier | study does not meet criteria for tier 1 or tier 3 | Example 2 | | ++ | - | ++ | + | - | - | + | ++ | - |
|  |  | Example 3 | | + | - | - | - | - | ++ | -- | + | + |
|  |  | Example 4 | | - | - | - | + | + | - | + | - | + |
| 3rd tier | “probably high” or “definitely high” risk of bias for all key criteria  AND  “definitely high” or “probably high” risk of bias for more than half of the other applicable criteria |  |  |  |  |  |  |  |  |  |  |  |
|  |  | Example 5 | | -- | - | - | - | + | - | + | - | - |
|  |  |  |  |  |  |  |  |  |  |  |  |  |

**1** National Toxicology Program (NTP), 2015a. Handbook for conducting a literature-based health assessment using OHAT approach for systematic review and evidence integration. Office of Health Assessment and Translation. <http://ntp.niehs.nih.gov/go/38673>.
