# Supplementary material for: Biological and health-related effects of weak static magnetic fields (≤ 1 mT) in humans and vertebrates: A systematic review
Source: PLoS One. 2020 Jun 9;15(6):e0230038. doi: 10.1371/journal.pone.0230038 (PMC7282627; doi:10.1371/journal.pone.0230038)
Supplement: S2 Table — (DOCX) [file pone.0230038.s003.DOCX]

**Excluded articles**

**Table A.1:** Excluded articles after full text screening (eligibility)

| **Number** | **Reference** | **Summary comment for exclusion** |
| --- | --- | --- |
|  | Abdel-Kader HM, Aref MI, Hussein FA. Coating failure of commercial orthodontic magnets and DNA fragmentation of oral mucosa cells. Aust Orthod J. 2008;24(1): 32-40. | magnetic flux density not provided |
|  | Abreu MC, Ponzoni D, Langie R, Artuzi FE, Puricelli E. Effects of a buried magnetic field on cranial bone reconstruction in rats. J Appl Oral Sci. 2016;24(2): 162-170. doi: 10.1590/1678-775720150336. | > 1 mT |
|  | Aguila J, Cudeiro J, Rivadulla C. Effects of Static Magnetic Fields on the Visual Cortex: reversible Visual Deficits and Reduction of Neuronal Activity. Cereb Cortex. 2016;26(2): 628-638. doi: 10.1093/cercor/bhu228. | > 1 mT |
|  | Akoyunoglou G. Effect of a magnetic field on carboxydismutase. Nature. 1964;202: 452-454. doi: 10.1038/202452a0. | other experimental study (in vitro) |
|  | Alfano AP, Taylor AG, Foresman PA, Dunkl PR, McConnell GG, et al. Static magnetic fields for treatment of fibromyalgia: a randomized controlled trial. J Altern Complement Med. 2004;7(1): 53-64. doi: 10.1089/107555301300004538. | magnetic flux density of control group not provided |
|  | Amara S, Abdelmelek H, Garrel C, Guiraud P, Douki T, Ravanat JL, et al. Zinc supplementation ameliorates static magnetic field-induced oxidative stress in rat tissues. Environ Toxicol Pharmacol. 2007;23(2): 193-197. doi: 10.1016/j.etap.2006.09.001. | > 1 mT |
|  | Anisimov VN, Zhukova OV, Beniashvili DS, Bilanishvili VG, Menabde MZ, Gupta D. Effect of the Light Regime and Electromagnetic Fields on Carcinogenesis of the Mammary Gland in Female Rats. Biophysics. 1996;41(4): 817-823. | exposure condition unclear |
|  | Anosov VN, Trukhan EM. A new approach to the problem of weak magnetic fields: an effect on living objects. Dokl Biochem Biophys. 2003;392: 274-278. doi: 10.1023/a:1026147031461. | other experimental study (in vitro) |
|  | Antal M, Laszlo J. Exposure to inhomogeneous static magnetic field ceases mechanical allodynia in neuropathic pain in mice. Bioelectromagnetics. 2009;30(6): 438-445. doi: 10.1002/bem.20498. | > 1 mT |
|  | Arias P, Adán-Arcay L, Puerta-Catoira B, Madrid A, Cudeiro J. Transcranial static magnetic field stimulation of M1 reduces corticospinal excitability without distorting sensorimotor integration in humans. Brain Stimul. 2017;10(2): 340-342. doi: 10.1016/j.brs.2017.01.002. | > 1 mT |
|  | Aydin N, Bezer M. The effect of an intramedullary implant with a static magnetic field on the healing of the osteotomised rabbit femur. Int Orthop. 2011;35(1): 135-141. doi: 10.1007/s00264-009-0932-9. | > 1 mT |
|  | Azanza MJ, del Moral A. Cell membrane biochemistry and neurobiological approach to biomagnetism. Prog Neurobiol. 1994;44(6): 517-601. doi: 10.1016/0301-0082(94)90004-3. | review, editorial, comment |
|  | Baker RR. Goal orientation by blindfolded humans after long-distance displacement: possible involvement of a magnetic sense. Science. 1980;210(4469): 555-557. doi: 10.1126/science.7423208. | (mechanisms of) magnetoreception |
|  | Bambini F, Santarelli A, Putignano A, Procaccini M, Orsini G, Di Iorio D, et al. Use of supercharged cover screw as static magnetic field generator for bone healing, 2nd part: in vivo enhancement of bone regeneration in rabbits. J Biol Regul Homeost Agents. 2017;31(2): 481-485. | > 1 mT |
|  | Barker AT, Cain MW. The claimed vasodilatory effect of a commercial permanent magnet foil: results of a double-blind trial. Clin Phys Physiol Meas. 1985;6(3): 261-263. doi: 10.1088/0143-0815/6/3/008. | > 1 mT |
|  | Barnothy JM, Barnothy MF. Second-day minimum in the growth curve of mice subjected to magnetic fields. Nature. 1963;200: 189. doi: 10.1038/200189a0. | > 1 mT |
|  | Barnothy JM. Growth rate of mice in static magnetic fields. Nature. 1963;200: 86-87. doi: 10.1038/200086a0. | > 1 mT |
|  | Barnothy MF, Barnothy JM. Magnetic fields and the number of blood platelets. Nature. 1970;225(5238): 1146-1147. doi: 10.1038/2251146a0. | > 1 mT |
|  | Barnothy MF, Sumegi I. Abnormalities in organs of mice induced by a magnetic field. Nature. 1969;221(5177): 270-271. | > 1 mT |
|  | Barregard L, Järvholm B, Ungethum E. Cancer among workers exposed to strong static magnetic fields. Lancet. 1985;326(8460): 892. doi: 10.1016/S0140-6736(85)90157-6. | > 1 mT |
|  | Bassett CA. Effects of a static magnetic field on fracture healing. Clin Orthop Relat Res. 1988;(234): 311-312. | review, editorial, comment |
|  | Baute V, Keskinyan VS, Sweeney ER, Bowden KD, Gordon A, Hutchens J, et al. A randomized, controlled trial of magnetic therapy for carpal tunnel syndrome. Muscle Nerve. 2018;58(2): 310-313. doi: 10.1002/mus.26120. | > 1 mT |
|  | Beaugrand JP. An attempt to confirm magnetic sensitivity in the pigeon, *Columba livia*. J Comp Physiol. 1976;110(3): 343-355. doi: 10.1007/BF00659150. | exposure condition unclear |
|  | Behari J, Mathur R. Exposure effects of static magnetic field on some physiological parameters of developing rats. Indian J Exp Biol. 1997;35(8): 894-897. | exposure condition unclear |
|  | Bekhite MM, Finkensieper A, Abou-Zaid FA, El-Shourbagy IK, El-Fiky NK, Omar KM, et al. Differential effects of high and low strength magnetic fields on mouse embryonic development and vasculogenesis of embryonic stem cells. Reprod Toxicol. 2016;65: 46-58. doi:10.1016/j.reprotox.2016.06.016. | magnetic flux density of control group not provided (remark: magnetic flux density of control group ≈ 0.04 µT (but not clear if alternating current magnetic field or static magnetic field (SMF) component) |
|  | Bell GB, Marino AA, Chesson AL. Alterations in brain electrical activity caused by magnetic fields: detecting the detection process. Electroencephalogr Clin Neurophysiol. 1992;83(6): 389-397. doi: 10.1016/0013-4694(92)90075-s. | > 1 mT |
|  | Bellossi A, Toujas L. The effect of a static uniform magnetic field on mice. A study of a Lewis tumour graft. Radiat Environ Biophys. 1982;20(2): 153-157. doi: 10.1007/bf01323936. | > 1 mT |
|  | Bellossi A. No effect of a static uniform magnetic field on mouse trypanosomiasis. Radiat Environ Biophys. 1983;22(4): 311-313. doi: 10.1007/bf01323681. | > 1 mT |
|  | Bellossi A. The effect of a static non-uniform magnetic field on mice. A study of Lewis tumour graft. Radiat Environ Biophys. 1986;25(3): 231-234. doi: 10.1007/bf01221230. | > 1 mT |
|  | Bellossi A. The effect of a static uniform magnetic field on mice a study of methylcholanthren carcinogenesis. Radiat Environ Biophys. 1984;23(2): 107-109. doi: 10.1007/BF01213734. | > 1 mT |
|  | Bellossi A. The effect of a static uniform magnetic field on mice. A study of methylcholanthren carcinogenesis. Radiat Environ Biophys. 1984;23(2): 107-109. doi: 10.1007/bf01213734. | > 1 mT |
|  | Beniashvili DS, Bilanishvili VG, Menabde MZ. Low-frequency electromagnetic radiation enhances the induction of rat mammary tumors by nitrosomethyl urea. Cancer Lett. 1991;61(1): 75-79. doi: 10.1016/0304-3835(91)90079-w. | magnetic flux density not provided |
|  | Bergman J, Robertson JR, Elia G. Effects of a magnetic field on pelvic floor muscle function in women with stress urinary incontinence. Altern Ther Health Med. 2004;10(3): 70-72. | magnetic flux density not provided |
|  | Bernhold M, Bondemark L. A magnetic appliance for treatment of snoring patients with and without obstructive sleep apnea. Am J Orthod Dentofacial Orthop. 1998;113(2): 144-155. doi: 10.1016/s0889-5406(98)70286-0. | magnetic flux density not provided |
|  | Blechman AM, Steger ER. Further comment on static magnetic field bioeffects. Am J Orthod Dentofacial Orthop. 1993;104(6): 20A-22A. doi: 10.1016/S0889-5406(05)80429-9. | review, editorial, comment |
|  | Bondemark L, Kurol J, Larsson A. Long-term effects of orthodontic magnets on human buccal mucosa--a clinical, histological and immunohistochemical study. Eur J Orthod. 1998;20(3): 211-218. doi: 10.1093/ejo/20.3.211. | > 1 mT |
|  | Bondemark L. Orthodontic magnets. A study of force and field pattern, biocompatibility and clinical effects. Swed Dent J Suppl. 1994;99: 1-148. | other experimental study (in vitro) |
|  | Bookman MA. Sensitivity of the homing pigeon to an earth-strength magnetic field. Nature. 1977;267(5609): 340-342. doi: 10.1038/267340a0. | (mechanisms of) magnetoreception |
|  | Borsa PA, Liggett CL. Flexible magnets are not effective in decreasing pain perception and recovery time after muscle microinjury. J Athl Train. 1988;33(2): 150-155. | > 1 mT |
|  | Boström JE, Fransson T, Henshaw I, Jakobsson S, Kullberg C, Åkesson S. Autumn migratory fuelling: a response to simulated magnetic displacements in juvenile wheatears, *Oenanthe oenanthe*. Behav Ecol Sociobiol. 2010;64(11): 1725-1732. doi: 10.1007/s00265-010-0985-1. | (mechanisms of) magnetoreception |
|  | Bowman JD, Thomas DC, London SJ, Peters JM. Hypothesis: the risk of childhood leukemia is related to combinations of power-frequency and static magnetic fields. Bioelectromagnetics. 1995;16(1): 48-59. doi: 10.1002/bem.2250160111. | co-exposure |
|  | Brix G, Strieth S, Strelczyk D, Dellian M, Griebel J, Eichhorn ME, et al. Static Magnetic Fields Affect Capillary Flow of Red Blood Cells in Striated Skin Muscle. Microcirculation. 2008;15(1): 15-26. doi: 10.1080/10739680701410850. | > 1 mT |
|  | Brown Jr FA, Scow KM. Magnetic induction of a circadian cycle in hamsters. J Interdiscipl Cycle Res. 1978;9(2): 137-145. doi: 10.1080/09291017809359632. | magnetic flux density of control group not provided |
|  | Budinger TF, Bristol KS, Yen CK, Wong P. Biological Effects of Static Magnetic Fields. Proceedings of the International Society for Magnetic Resonance in Medicine. 1984; Suppl 1: 113-114. | not peer-reviewed, exposure condition unclear |
|  | Buettner UW. Human interactions with ultra high fields. Ann N Y Acad Sci. 1992;649: 59-66. doi: 10.1111/j.1749-6632.1992.tb49596.x. | magnetic resonance imaging (MRI) |
|  | Bunch KJ, Swanson J, Vincent TJ, Murphy MF. Magnetic fields and childhood cancer: an epidemiological investigation of the effects of high-voltage underground cables. J Radiol Prot. 2015;35(3): 695-705. doi: 10.1088/0952-4746/35/3/695. | magnetic flux density not provided |
|  | Calcagnotto T, Schwengber MMB, De Antoni CC, de Oliveira DL, Vago TM, Guilinelli J. Magnetic Field Effects on Bone Repair after Calcium Phosphate Cement Implants: Histometric and Biochemistry Evaluation. Ann Maxillofac Surg. 2017;7(1): 18-24. doi: 10.4103/ams.ams_2_17. | > 1 mT, co-exposure (cement implant) |
|  | Camilleri S, McDonald F. Static magnetic field effects on the sagittal suture in Rattus norvegicus. Am J Orthod Dentofacial Orthop. 1993;103(3): 240-246. doi: 10.1016/0889-5406(93)70004-8. | > 1 mT |
|  | Carpenter JS, Wells N, Lambert B, Watson P, Slayton T, Chak B, et al. A pilot study of magnetic therapy for hot flashes after breast cancer. Cancer Nurs. 2002;25(2): 104-109. doi: 10.1097/00002820-200204000-00005. | magnetic flux density not provided |
|  | Carrasco-López C, Soto-León V, Céspedes V, Profice P, Strange BA, Foffani G, et al. Static Magnetic Field Stimulation over Parietal Cortex Enhances Somatosensory Detection in Humans. J Neurosci. 2017;37(14): 3840-3847. doi: 10.1523/JNEUROSCI.2123-16.2017. | > 1 mT |
|  | Caselli MA, Clark N, Lazarus S, Velez Z, Venegas L. Evaluation of Magnetic Foil and PPT Insoles in the Treatment of Heel Pain. J Am Podiatr Med Assoc. 1997;87(1): 11-16. doi: 10.7547/87507315-87-1-11. | > 1 mT |
|  | Caswell JM, Singh M, Persinger MA. Simulated sudden increase in geomagnetic activity and its effect on heart rate variability: Experimental verification of correlation studies. Life Sci Space Res (Amst). 2016;10: 47-52. doi: 10.1016/j.lssr.2016.08.001. | geomagnetic storm |
|  | Celik O, Ugras M, Hascalik S, Aydin NE, Abbasov T. Enhanced endometrial response to a magnetic intrauterine device: A preliminary study. Eur J Contracept Reprod Health Care. 2009;14(6): 437-443. doi: 10.3109/13625180903318259. | > 1 mT |
|  | Cepeda MS, Carr DB, Sarquis T, Miranda N, Garcia RJ, Zarate C. Static magnetic therapy does not decrease pain or opioid requirements: a randomized double-blind trial. Anesth Analg. 2007;104(2): 290-294. doi: 10.1213/01.ane.0000230613.25754.08. | > 1 mT |
|  | Cerny R. The reaction of dental tissues to magnetic fields. Aust Dent J. 1980;25(5): 264-268. doi: 10.1111/j.1834-7819.1980.tb05198.x. | > 1 mT |
|  | Chae KS, Oh IT, Lee SH, Kim SC. Blue light-dependent human magnetoreception in geomagnetic food orientation. PLoS One. 2019;14(2): e0211826. doi: 10.1371/journal.pone.0211826. | (mechanisms of) magnetoreception |
|  | Choleris E, Del Seppia C, Thomas AW, Luschi P, Ghione G, Moran GR, et al. Shielding, but not zeroing of the ambient magnetic field reduces stress-induced analgesia in mice. Proc Biol Sci. 2002;269(1487): 193-201. doi: 10.1098/rspb.2001.1866. | field deprivation/hypomagnetic field |
|  | Coballase-Urrutia E, Navarro L, Ortiz JL, Verdugo-Díaz L, Gallardo JM, Hernández ME, Estrada-Rojo F. Static Magnetic Fields Modulate the Response of Different Oxidative Stress Markers in a Restraint Stress Model Animal. Biomed Res Int. 2018: 3960408. doi: 10.1155/2018/3960408. | exposure condition unclear |
|  | Coles R, Bradley P, Donaldson I, Dingle A. A trial of tinnitus therapy with ear-canal magnets. Clin Otolaryngol Allied Sci. 1991;16(4): 371-372. doi: 10.1111/j.1365-2273.1991.tb02072.x. | > 1 mT |
|  | Collacott EA, Zimmerman JT, White DW, Rindone JP. Bipolar permanent magnets for the treatment of chronic low back pain: a pilot study. JAMA. 2000;283(10): 1322-1325. doi: 10.1001/jama.283.10.1322. | > 1 mT |
|  | Costantino C, Pogliacomi F, Passera F, Concari G. Treatment of wrist and hand fractures with natural magnets: preliminary report. Acta Biomed. 2007;78(3): 198-203. | > 1 mT |
|  | Creim JA, Lovely RH, Miller DL, Anderson LE. Rats can discriminate illuminance, but not magnetic fields, as a stimulus for learning a two-choice discrimination. Bioelectromagnetics. 2002;23(7): 545-549. doi: 10.1002/bem.10052. | co-exposure, magnetic flux density of control group not provided |
|  | Darendeliler MA, Darendeliler A, Sinclair PM. Effects of static magnetic and pulsed electromagnetic fields on bone healing. Int J Adult Orthodon Orthognath Surg. 1997;12(1): 43-53. | > 1 mT |
|  | Darendeliler MA, Sinclair PM, Kusy RP. The effects of samarium-cobalt magnets and pulsed electromagnetic fields on tooth movement. Am J Orthod Dentofacial Orthop. 1995;107(6): 578-588. doi: 10.1016/s0889-5406(95)70100-1. | exposure condition unclear, magnetic flux density of control group not provided |
|  | De Luka SR, Ilic AZ, Jankovic S, Djordjevich DM, Cirkovic S, Milovanovich ID, et al. Subchronic exposure to static magnetic field differently affects zinc and copper content in murine organs. Int J Radiat Biol. 2016;9: 140-147. doi: 10.3109/09553002.2016.1135266. | > 1 mT |
|  | Denegre JM, Valles Jr JM, Lin K, Jordan WB, Mowry KL. Cleavage planes in frog eggs are altered by strong magnetic fields. Proc Natl Acad Sci U S A. 1998;95(25): 14729-14732. | > 1 mT |
|  | Deutschlander ME, Freake MJ, Borland SC, Phillips JB, Madden RC, Anderson LE, et al. Learned magnetic compass orientation by the Siberian hamster, *Phodopus sungorus*. Anim Behav. 2003;65(4): 779-786. doi: 10.1006/anbe.2003.2111. | (mechanisms of) magnetoreception |
|  | Dileone M, Carrasco-López MC, Segundo-Rodriguez JC, Mordillo-Mateos L, López-Ariztegui N, Alonso-Frech F, et al. Dopamine-dependent changes of cortical excitability induced by transcranial static magnetic field stimulation in Parkinson's disease. Sci Rep. 2017;7(1): 4329. doi: 10.1038/s41598-017-04254-y. | > 1 mT |
|  | Dileone M, Mordillo-Mateos L, Oliviero A, Foffani G. Long-lasting effects of transcranial static magnetic field stimulation on motor cortex excitability. Brain Stimul. 2018;11(4): 676-688. doi: 10.1016/j.brs.2018.02.005. | magnetic flux density not provided |
|  | Dobson J, St Pierre T, Wieser HG, Fuller M. Changes in paroxysmal brainwave patterns of epileptics by weak-field magnetic stimulation. Bioelectromagnetics. 2000;21(2): 94-99. doi: 10.1002/(sici)1521-186x(200002)21:2<94::aid-bem3>3.0.co;2-7. | magnetic flux density of control group not provided |
|  | Dobson J, St Pierre TG, Schultheiss-Grassi PP, Wieser HG, Kuster N. Analysis of EEG data from weak-field magnetic stimulation of mesial temporal lobe epilepsy patients. Brain Res. 2000:868(2): 386-391. doi: 10.1016/s0006-8993(00)02422-7. | > 1 mT |
|  | Duda D, Grzesik J, Pawlicki K. Changes in liver and kidney concentration of copper, manganese, cobalt and iron in rats exposed to static and low-frequency (50 Hz) magnetic fields. J Trace Elem Electrolytes Health Dis. 1991;5(3): 181-186. | > 1 mT |
|  | Easterly CE. Cardiovascular risk from exposure to static magnetic fields. Am Ind Hyg Assoc J. 1982;43(7): 533-539. doi: 10.1080/15298668291410161. | other experimental study (simulation) |
|  | Eccles N. Static magnets prevent leg ulcer recurrence: savings for the NHS? Br J Community Nurs. 2006;11(Sup1): S26-S30. doi: 10.12968/bjcn.2006.11.Sup1.20595. | magnetic flux density not provided |
|  | Eccles NK, Hollinworth H. A pilot study to determine whether a static magnetic device can promote chronic leg ulcer healing. J Wound Care. 2005;14(2): 64-67. doi: 10.12968/jowc.2005.14.2.26731. | > 1 mT |
|  | Ekici Y, Aydogan C, Balcik C, Haberal N, Kirnap M, Moray G, et al. Effect of static magnetic field on experimental dermal wound strength. Indian J Plast Surg. 2012;45(2): 215-219. doi: 10.4103/0970-0358.101281. | > 1 mT |
|  | Elbers D, Bulte M, Bairlein F, Mouritsen H, Heyers D. Magnetic activation in the brain of the migratory northern wheatear (*Oenanthe oenanthe*). J Comp Physiol A Neuroethol Sens Neural Behav Physiol. 2017;203(8): 591-600. doi: 10.1007/s00359-017-1167-7. | (mechanisms of) magnetoreception |
|  | El'darov AL, Kholodov YA. Effect of constant magnetic field on motor activity of birds. Fed Proc Transl Suppl. 1965;24(3): 431-433. | (mechanisms of) magnetoreception, magnetic flux density of control group not provided, exposure condition unclear |
|  | el-Messiery MA. Magnetic field interaction with streaming potentials in cancellous bone. Biomaterials. 1992;13(3): 168-171. doi: 10.1016/0142-9612(92)90066-w. | other experimental study (in vitro) |
|  | Esformes I, Kummer FJ, Livelli TJ. Biological effects of magnetic fields generated with CoSm magnets. Bull Hosp Jt Dis Orthop Inst. 1981;41: 81-87. | > 1 mT |
|  | Feinendegen LE, Mühlensiepen H. Effect of static magnetic field on cellular metabolism in the living mouse. Endeavour. 1988;12(3): 119-123. doi: 10.1016/0160-9327(88)90132-9. | > 1 mT |
|  | Fey DP, Jakubowska M, Greszkiewicz M, Andrulewicz E, Otremba Z, Urban-Malinga B. Are magnetic and electromagnetic fields of anthropogenic origin potential threats to early life stages of fish? Aquat Toxicol. 2019;209: 150-158. doi: 10.1016/j.aquatox.2019.01.023. | > 1 mT |
|  | Fitak RR, Schweikert LE, Wheeler BR, Ernst DA, Lohmann KJ, Johnsen S. Near absence of differential gene expression in the retina of rainbow trout after exposure to a magnetic pulse: implications for magnetoreception. Biol Lett. 2018;14(6): pii: 20180209. doi: 10.1098/rsbl.2018.0209. | > 1 mT |
|  | Formicki K, Sadowski M, Korzelecka-Orkisz A, Winnicki A. Behaviour of trout (*Salmo trutta* L*.*) larvae and fry in a constant magnetic field. J Appl Ichthyol. 2004;20(4): 290-294. doi: 10.1111/j.1439-0426.2004.00556.x. | (mechanisms of) magnetoreception |
|  | Formicki K, Tanski A, Sadowski M, Winnicki A. Effects of magnetic fields on fyke net performance J Appl Ichthyol. 2004;20(5): 402-406. doi: 10.1111/j.1439-0426.2004.00568.x. | (mechanisms of) magnetoreception |
|  | Formicki K, Tanski A, Winnicki A. Effects of magnetic field on the direction of fish movement under natural conditions. 2002; 27th General Assembly, URSI 2002, Maastricht, The Netherlands: K2.O.5(842), 1-3. | (mechanisms of) magnetoreception, not peer-reviewed |
|  | Formicki K, Tanski A, Winnicki A. Preliminary results of studies on magnetic field effects on semi-circular fyke net catch potential. Folia Universitatis Agriculturae Stetinensis. Piscaria. 2000;27: 69-74. | (mechanisms of) magnetoreception |
|  | Formicki K, Winnicki A. Reaction of fish embryos and larvae to constant magnetic fields. Ital J Zool. 1998;65: 479-482. doi: 10.1080/11250009809386870. | magnetic flux density of control group not provided |
|  | Formicki K. Respiratory movements of trout (*Salmo trutta* L.) larvae during exposure to magnetic field. AIeP. 1992;22(2): 149-154. doi: 10.3750/AIP1992.22.2.08. | > 1 mT |
|  | Fransson T, Jakobsson S, Johansson P, Kullberg C, Lind J, Vallin A. Magnetic cues trigger extensive refuelling. Nature. 2001;414(6859): 35-36. doi: 10.1038/35102115. | (mechanisms of) magnetoreception |
|  | Freire R, Birch TE. Conditioning to magnetic direction in the Pekin duck (*Anas platyrhynchos domestica*). J Exp Biol. 2010;213: 3423-3426. doi: 10.1242/jeb.047613. | (mechanisms of) magnetoreception |
|  | Fuller M, Dobson J, Wieser HG, Moser S. On the sensitivity of the human brain to magnetic fields: evocation of epileptiform activity. Brain Res Bull. 1995;36(2): 155-159. doi: 10.1016/0361-9230(94)00183-2. | > 1 mT |
|  | Fuller M, Wilson CL, Velasco AL, Dunn JR, Zoeger J. On the confirmation of an effect of magnetic fields on the interictal firing rate of epileptic patients. Brain Res Bull. 2003;60(1-2): 43-52. doi: 10.1016/s0361-9230(03)00027-3. | > 1 mT |
|  | Funk RH, Knels L, Augstein A, Marquetant R, Dertinger HF(2014): Potent stimulation of blood flow in fingers of volunteers after local short-term treatment with low-frequency magnetic fields from a novel device. Evid Based Complement Alternat Med. 2014: 543564. doi: 10.1155/2014/543564. | > 1 mT |
|  | Geng Y, Zhang X. EEG EPs analysis of magnetic stimulation on acupoint of Shenmen(HT7). In: 2012 Annual International Conference of the IEEE Engineering in Medicine and Biology Society; 2012 Aug 28 – Sep 1; San Diego, CA, USA; 2012. p. 5745-5748. doi: 10.1109/EMBC.2012.6347299. | > 1 mT |
|  | Gerardi G, De Ninno A, Ferrari V, Mazzariol S, Bernardini D, Segato S. The Effect of Electromagnetic Fields with the Mg2+ Cyclotron Frequency on Mouse Reproductive Performance. J Electromagn Anal. 2016;8(3): 115-123. doi: 10.4236/jemaa.2016.87012. | no static magnetic field (SMF) |
|  | Gmitrova A, Gmitrov J. Effect of a Permanent Magnetic Field on Blood Pressure Regulation. J Bioelectricity. 1990;9(1): 79-83. doi: 10.3109/15368379009027762. | > 1 mT |
|  | Gorczynska E, Galka G, Wegrzynowicz R, Mikosza H. Effect of magnetic field on the process of cell respiration in mitochondria of rats. Physiol Chem Phys Med NMR. 1986;18(1): 61-69. | > 1 mT |
|  | Gorczynska E, Wegrzynowicz R. Activity of acid and alkali phosphatase in guinea pigs exposed to the static magnetic fields. J Hyg Epidemiol Microbiol Immunol. 1985;29(2): 135-139. | > 1 mT |
|  | Gorczynska E, Wegrzynowicz R. Glucose homeostasis in rats exposed to magnetic fields. Invest Radiol. 1991;26(12): 1095-1100. doi: 10.1097/00004424-199112000-00013. | magnetic flux density of control group not provided |
|  | Gorczynska E, Wegrzynowicz R. Structural and functional changes in organelles of liver cells in rats exposed to magnetic fields. Environ Res. 1991;55(2): 188-198. doi: 10.1016/s0013-9351(05)80175-6. | magnetic flux density of control group not provided |
|  | Gorczynska E. A magnetic field generates morphological changes in guinea pig bone marrow. Folia Morphol (Praha). 1987;35(1): 40-45. | > 1 mT |
|  | Gould JS, Able KP. Human homing: an elusive phenomenon. Science. 1981;212(4498): 1061-1063. doi: 10.1126/science.7233200. | (mechanisms of) magnetoreception, magnetic flux density not provided |
|  | Grande DA, Magee FP, Weinstein AM, McLeod BR. The effect of low-energy combined AC and DC magnetic fields on articular cartilage metabolism. Ann N Y Acad Sci. 1991;635: 404-407. doi: 10.1111/j.1749-6632.1991.tb36510.x. | other experimental study (in vitro) |
|  | Gray JR, Frith CH, Parker JD. In vivo enhancement of chemotherapy with static electric or magnetic fields. Bioelectromagnetics. 2000;21(8): 575-583. doi: 10.1002/1521-186X(200012)21:8<575::AID-BEM3>3.0.CO;2-F. | > 1 mT |
|  | Grzesik J, Bortel M, Duda D, Kuska R, Ludyga K, Michnik J, et al. Influence of a static magnetic field on the reproductive function, certain biochemical indices and behaviour of rats. Pol J Occup Med. 1988;1(4): 329-339. | > 1 mT |
|  | Gujjalapudi M, Anam C, Mamidi P, Chiluka R, Kumar AG, Bibinagar R. Effect of Magnetic Field on Bone Healing around Endosseous Implants - An In-vivo Study. J Clin Diagn Res. 2016;10(10): ZF01-ZF04. doi: 10.7860/JCDR/2016/21509.8666. | > 1 mT |
|  | Gurfinkel YI, Vasin AL, Pishchalnikov RY, Sarimov RM, Sasonko ML, Matveeva TA. Geomagnetic storm under laboratory conditions: randomized experiment. Int J Biometeorol. 2018;62(4): 501-512. doi: 10.1007/s00484-017-1460-8. | geomagnetic storm |
|  | Gurhan H, Bruzón R, Xiong Y, Barnes F. Effect of a low intensity static magnetic field on different biological parameters that characterize the cellular stress. In: 2018 United States National Committee of URSI National Radio Science Meeting (USNC-URSI NRSM); 2018 Jan 4-7; Boulder, CO, USA. 2002. p. 1-2. | other experimental study (in vitro) |
|  | Hansson Mild K, Sandström M, Lovtrup S. Development of Xenopus laevis embryos in a static magnetic field. Bioelectromagnetics. 1981;2(2): 199-201. doi: 10.1002/bem.2250020210. | > 1 mT |
|  | Harlow T, Greaves C, White A, Brown L, Hart A, Ernst E. Randomised controlled trial of magnetic bracelets for relieving pain in osteoarthritis of the hip and knee. BMJ. 2004;329(7480): 1450-1454. doi: 10.1136/bmj.329.7480.1450. | > 1 mT |
|  | Hashish AH, El-Missiry MA, Abdelkader HI, Abou-Saleh RH. Assessment of biological changes of continuous whole body exposure to static magnetic field and extremely low frequency electromagnetic fields in mice. Ecotoxicol Environ Saf. 2008;71(3): 895-902. doi: 10.1016/j.ecoenv.2007.10.002. | magnetic flux density of control group not provided |
|  | Haupt RC, Nolfi JR. The effects of high voltage transmission lines on the health of adjacent resident populations. Am J Public Health. 1984;74(1): 76-78. doi: 10.2105/ajph.74.1.76. | magnetic flux density not provided |
|  | He Y, Sun W, Leung PS, Chow YT. Effect of Static Magnetic Field of Electric Vehicles on Driving Performance and on Neuro-Psychological Cognitive Functions. Int J Environ Res Public Health. 2019;16(18): pii: E3382. doi: 10.3390/ijerph16183382. | magnetic flux density of control group not provided |
|  | Heimrath K, Spröggel A, Repplinger S, Heinze HJ, Zaehle T. Transcranial Static Magnetic Field Stimulation Over the Temporal Cortex Modulating the Right Ear Advantage in Dichotic Listening. Neuromodulation. Forthcoming 2019. doi: 10.1111/ner.13023. | > 1 mT |
|  | Henshaw I, Fransson T, Jakobsson S, Jenni-Eiermann S, Kullberg C. Information from the geomagnetic field triggers a reduced adrenocortical response in a migratory bird. J Exp Biol. 2009;212: 2902-2907. doi: 10.1242/jeb.033332. | (mechanisms of) magnetoreception |
|  | Heyers D, Zapka M, Hoffmeister M, Wild JM, Mouritsen H. Magnetic field changes activate the trigeminal brainstem complex in a migratory bird. Proc Natl Acad Sci U S A. 2010;107(20): 9394-9399. doi: 10.1073/pnas.0907068107. | (mechanisms of) magnetoreception |
|  | Hinman MR, Ford J, Heyl H. Effects of static magnets on chronic knee pain and physical function: a double-blind study. Altern Ther Health Med. 2002;8(4): 50-55. | > 1 mT |
|  | Hinman MR. Comparative effect of positive and negative static magnetic fields on heart rate and blood pressure in healthy adults. Clin Rehabil. 2002;16(6): 669-674. doi: 10.1191/0269215502cr538oa. | > 1 mT |
|  | Hirai T, Taniura H, Goto Y, Ogura M, Sng JC, Yoneda Y. Stimulation of ubiquitin-proteasome pathway through the expression of amidohydrolase for N-terminal asparagine (Ntan1) in cultured rat hippocampal neurons exposed to static magnetism. J Neurochem. 2006;96(6): 1519-1530. doi: 10.1111/j.1471-4159.2006.03655.x. | other experimental study (in vitro), > 1 mT |
|  | Holcomb RR, Parker RA, Harrison MS. Biomagnetics in the treatment of human pain - past, present, future. Environ Med. 1991;8(2): 24-30. | > 1 mT |
|  | Holland RA, Thorup K, Vonhof MJ, Cochran WW, Wikelski M. Bat orientation using earth's magnetic field. Nature. 2006;444(7119): 702. doi: 10.1038/444702a. | (mechanisms of) magnetoreception |
|  | Hong CZ, Lin JC, Bender LF, Schaeffer JN, Meltzer RJ, Causin P. Magnetic necklace: its therapeutic effectiveness on neck and shoulder pain. Arch Phys Med Rehabil. 1982;63(10): 462-466. | > 1 mT |
|  | Ilieva M, Bianco G, Akesson S. Does migratory distance affect fuelling in a medium-distance passerine migrant?: results from direct and step-wise simulated magnetic displacements. Biol Open. 2016; 5(3): 272-278. doi: 10.1242/bio.014779. | (mechanisms of) magnetoreception |
|  | Jankovic BD, Jovanova-Nesic K, Nikolic V, Nikolic P. Brain-applied magnetic fields and immune response: role of the pineal gland. Int J Neurosci. 1993;70(1-2): 127-134. doi: 10.3109/00207459309000568. | > 1 mT |
|  | Jankovic BD, Nikolic P, Cupic V, Hladni K. Potentiation of immune responsiveness in aging by static magnetic fields applied to the brain. Role of the pineal gland. Ann N Y Acad Sci. 1994;719: 410-418. doi: 10.1111/j.1749-6632.1994.tb56846.x. | > 1 mT |
|  | Jasmi VK, Samadi F, Eimani H, Hasani S, Fathi R, Shahverdi A. Follicle Development in Grafted Mouse Ovaries after Vitrification Processes Under Static Magnetic Field. Cryo Letters. 2017;38(3): 166-177. | other experimental study (in vitro) |
|  | Jovanova-Nesic K, Skokljev AA. Magnetic brain stimulation and immune response in the rat with lesioned brain structures. Acupunct Electrother Res. 1990;15(1): 27-35. doi: 10.3727/036012990816358289. | > 1 mT |
|  | Kanai S, Taniguchi N. Efficacy of static magnetic field for pain of adjuvant arthritis rats. Adv Biosci Biotechnol. 2012;3(4A): 511-515. doi: 10.4236/abb.2012.324067. | > 1 mT |
|  | Kazemein Jasemi VS, Samadi F, Eimani H, Hasani S, Fathi R, Shahverdi A. Comparison of Allotransplantation of Fresh and Vitrified Mouse Ovaries to The Testicular Tissue under Influence of The Static Magnetic Field. Cell J. 2017;19(3): 492-505. doi: 10.22074/cellj.2017.4513. | magnetic flux density of control group not provided |
|  | Kazemein Jasemi VS, Samadi F, Eimani H, Hasani S, Fathi R, Shahverdi A, et al. Function of vitrified mouse ovaries tissue under static magnetic field after autotransplantation. Vet Res Forum. 2017;8(3): 243-249. | other experimental study (in vitro) |
|  | Kelleher MO, Al-Abri RK, Lenihan DV, Glasby MA. Use of a static magnetic field to promote recovery after peripheral nerve injury. J Neurosurg. 2006;105(4): 610-615. doi: 10.3171/jns.2006.105.4.610. | > 1 mT |
|  | Khan MW, Roivainen P, Herrala M, Tiikkaja M, Sallmén M, Hietanen M, et al. A pilot study on the reproductive risks of maternal exposure to magnetic fields from electronic article surveillance systems. Int J Radiat Biol. 2018;94(10): 902-908. doi: 10.1080/09553002.2018.1439197. | no SMF |
|  | Kilfoyle AK, Jermain RF, Dhanak MR, Huston JP, Spieler RE. Effects of EMF emissions from undersea electric cables on coral reef fish. Bioelectromagnetics. 2018;39(1): 35-52. doi: 10.1002/bem.22092. | field deprivation/hypomagnetic field, magnetic flux density of control group not provided |
|  | Kim EC, Leesungbok R, Lee SW, Hong JY, Ko EJ, Ahn SJ. Effects of static magnetic fields on bone regeneration of implants in the rabbit: micro-CT, histologic, microarray, and real-time PCR analyses. Clin Oral Implants Res. 2017;28(4): 396-405. doi: 10.1111/clr.12812. | > 1 mT |
|  | Kim TS. Relation of magnetic field therapy to pain and power over time in persons with chronic primary headache: a pilot study. Visions. 2001;9(1): 27-42. | > 1 mT |
|  | Kirimoto H, Tamaki H, Matsumoto T, Sugawara K, Suzuki M, Oyama M, et al. Effect of transcranial static magnetic field stimulation over the sensorimotor cortex on somatosensory evoked potentials in humans. Brain Stimul. 2014:7(6): 836-840. doi: 10.1016/j.brs.2014.09.016. | > 1 mT |
|  | Kirimoto H, Tamaki H, Otsuru N, Yamashiro K, Onishi H, Nojima I, et al. Transcranial Static Magnetic Field Stimulation over the Primary Motor Cortex Induces Plastic Changes in Cortical Nociceptive Processing. Front Hum Neurosci. 2018;12: e63. doi: 10.3389/fnhum.2018.00063. | > 1 mT |
|  | Kirschvink JL, Kuwajima T, Ueno S, Kirschvink SJ, Diaz-Ricci J, Morales A, et al. Discrimination of low-frequency magnetic fields by honeybees: biophysics and experimental tests. Soc Gen Physiol Ser. 1992;47: 225-240. | review, editorial, comment |
|  | Kishkinev D, Chernetsov N, Pakhomov A, Heyers D, Mouritsen H. Eurasian reed warblers compensate for virtual magnetic displacement. Curr Biol. 2015;25(19): R822-R824. doi: 10.1016/j.cub.2015.08.012. | (mechanisms of) magnetoreception |
|  | Kiss B, Gyires K, Kellermayer M, Laszlo JF. Lateral gradients significantly enhance static magnetic field-induced inhibition of pain responses in mice-a double blind experimental study. Bioelectromagnetics. 2013;34(5): 385-396. doi: 10.1002/bem.21781. | > 1 mT |
|  | Kiss B, Laszlo JF, Szalai A, Porszasz R(2015): Analysis of the Effect of Locally Applied Inhomogeneous Static Magnetic Field-Exposure on Mouse Ear Edema - A Double Blind Study. PLoS One. 2015;10(2): e0118089. doi: 10.1371/journal.pone.0118089. | > 1 mT |
|  | Klepetko W, Moritz A, Holle J, Laczkovics A. Zur Problematik der Narben nach Herzoperationen unter besonderer Berücksichtigung der Anwendung statischer Magnetfelder. Wien Klin Wochenschr. 1984;96(5): 185-190. | > 1 mT |
|  | Klimley AP, Wyman MT, Kavet R. Chinook salmon and green sturgeon migrate through San Francisco Estuary despite large distortions in the local magnetic field produced by bridges. PLoS One. 2017;12(6): e0169031. doi: 10.1371/journal.pone.0169031. | (mechanisms of) magnetoreception |
|  | Kloiber O, Okada Y, Hossmann KA. Keine Störung der elektrischen Aktivität des Gehirns der Katze in einem 4,7 T starken statischen Magnetfeld. Klin Neurophysiol. 1990;21(4): 229-232. doi: 10.1055/s-2008-1060798. | > 1 mT |
|  | Kobluk CN, Johnston GR, Lauper L. A Scintigraphic Investigation of Magnetic Field Therapy on the Equine Third Metacarpus. Vet Comp Orthop Traumatol. 1994;7(1): 14-18. doi: 10.1055/s-0038-1633036. | > 1 mT |
|  | Koziak AM, Desjardins D, Keenliside LD, Thomas AW, Prato FS. Light alters nociceptive effects of magnetic field shielding. Bioelectromagnetics. 2006;27(1): 10-15. doi: 10.1002/bem.20170. | field deprivation/hypomagnetic field, co-exposure (light) |
|  | Kreithen ML, Keeton WT. Attempts to condition homing pigeons to magnetic stimuli. J Comp Physiol. 1974;91(4): 355-362. doi: 10.1007/BF00694466. | (mechanisms of) magnetoreception |
|  | Krishnan V, Park SA, Shin SS, Alon L, Tressler CM, Stokes W, et al. Wireless control of cellular function by activation of a novel protein responsive to electromagnetic fields. Sci Rep. 2018;8(1): 8764. doi: 10.1038/s41598-018-27087-9. | > 1 mT |
|  | Krylov VV, Chebotareva YV, Izyumov YG. Delayed consequences of the influence of simulated geomagnetic storms on roach *Rutilus rutilus* embryos. J Fish Biol. 2019;95(6): 1422-1429. doi: 10.1111/jfb.14150. | geomagnetic storm |
|  | Kufner M, Brückner S, Kammer T. No modulatory effects by transcranial static magnetic field stimulation of human motor and somatosensory cortex. Brain Stimul. 2017;10(3): 703-710. doi: 10.1016/j.brs.2017.03.001. | > 1 mT |
|  | Kuipers NT, Sauder CL, Ray CA. Influence of static magnetic fields on pain perception and sympathetic nerve activity in humans. J Appl Physiol. 2007;102(4): 1410-1415. doi: 10.1152/japplphysiol.00734.2006. | > 1 mT |
|  | Kullberg C, Henshaw I, Jakobsson S, Johansson P, Fransson T. Fuelling decisions in migratory birds: geomagnetic cues override the seasonal effect. Proc Biol Sci. 2007;274(1622): 2145-2151. doi: 10.1098/rspb.2007.0554. | (mechanisms of) magnetoreception |
|  | Kullberg C, Lind J, Fransson T, Jakobsson S, Vallin A. Magnetic cues and time of season affect fuel deposition in migratory thrush nightingales (*Luscinia luscinia*). Proc Biol Sci. 2003;270(1513): 373-378. doi: 10.1098/rspb.2002.2273. | (mechanisms of) magnetoreception |
|  | Lacroix A, Proulx-Bégin L, Hamel R, De Beaumont L, Bernier PM, Lepage JF. Static magnetic stimulation of the primary motor cortex impairs online but not offline motor sequence learning. Sci Rep. 2019;9(1): 9886. doi: 10.1038/s41598-019-46379-2. | > 1 mT |
|  | Laforge H, Sadeghi MR, Seguin MK. Magnetostatic field effect: stress syndrome pattern and functional relation with intensity. J Psychol. 1986;120(3): 299-304. doi: 10.1080/00223980.1986.10545256. | data for specific exposure groups not provided |
|  | Lai WY, Huang YC, Chang WJ, Wang HT, Fong TH, Lin CT, Huang HM. Static magnetic field attenuates lipopolysaccharide-induced multiple organ failure: A histopathologic study in mice. Int J Radiat Biol. 2015;91(2): 135-141. doi: 10.3109/09553002.2015.959669. | > 1 mT |
|  | Laszlo J, Reiczigel J, Szekely L, Gasparics A, Bogar I, Bors L, Racz B, Gyires K. Optimization of static magnetic field parameters improves analgesic effect in mice. Bioelectromagnetics. 2007;28(8): 615-627. doi: 10.1002/bem.20341. | > 1 mT |
|  | Laszlo JF, Gyires K. Analysis of inhomogeneous static magnetic field-induced antinociceptive activity in mice. PIERS Online. 2010;6(4): 307-313. | > 1 mT |
|  | Lazarev AI, Siplivyi GV, Kukureka AV, Siplivaya LE. Effect of UV irradiation and magnetic field on immunometabolic effects of antibiotics immobilized in cell carriers. Bull Exp Biol Med. 2008;145(6): 717-720. doi: 10.1007/s10517-008-0167-6. | > 1 mT |
|  | Lazetic B, Pekaric-Nadj N. Heart Rate in Rats Exposed to Constant Magnetic Fields. Electro Magnetobiol. 1993;12(2): 117-123. doi: 10.3109/15368379309012866. | > 1 mT |
|  | Lerchl A, Nonaka KO, Reiter RJ. Pineal gland "magnetosensitivity" to static magnetic fields is a consequence of induced electric currents (eddy currents). J Pineal Res. 1991;10(3): 109-116. doi: 10.1111/j.1600-079X.1991.tb00826.x. | (mechanisms of) magnetoreception |
|  | Lerchl A, Nonaka KO, Stokkan KA, Reiter RJ. Marked rapid alterations in nocturnal pineal serotonin metabolism in mice and rats exposed to weak intermittent magnetic fields. Biochem Biophys Res Commun. 1990;169(1): 102-108. doi: 10.1016/0006-291X(90)91439-Y. | (mechanisms of) magnetoreception |
|  | Levengood WC. A new teratogenic agent applied to amphibian embryos. J Embryol Exp Morphol. 1969;21(1): 23-31. | > 1 mT |
|  | Li Z, Tam EW, Mak AF, Lau RY. Wavelet analysis of the effects of static magnetic field on skin blood flowmotion: investigation using an in vivo rat model. In Vivo. 2007;21(1): 61-68. | > 1 mT |
|  | Liburdy RP. Biological interactions of cellular systems with time-varying magnetic fields. Ann N Y Acad Sci. 1992;649(1): 74-95. doi: 10.1111/j.1749-6632.1992.tb49599.x. | other experimental study (in vitro), > 1 mT, co-exposure |
|  | Lin SL, Chang WJ, Lin YS, Ou KL, Lin CT, Lin CP, Huang HM. Static magnetic field attenuates mortality rate of mice by increasing the production of IL-1 receptor antagonist. Int J Radiat Biol. 2009;85(7): 633-640. doi: 10.1080/09553000902993908. | > 1 mT |
|  | Linder-Aronson A, Forsberg CM, Rygh P, Lindskog S. Tissue response to space closure in monkeys: a comparison of orthodontic magnets and superelastic coil springs. Eur J Orthod. 1996;18(6): 581-588. doi: 10.1093/ejo/18.6.581. | magnetic flux density not provided |
|  | Linder-Aronson A, Lindskog S, Rygh P. Orthodontic Magnets: Effects on Gingival Epithelium and Alveolar Bone in Monkeys. Eur J Orthod. 1992;14(4): 255-263. doi: 10.1093/ejo/14.4.255. | > 1 mT |
|  | Linder-Aronson A, Rygh P, Lindskog S. Effects of orthodontic magnets on cutaneous epithelial thickness and tibial bone growth in rats. Acta Odontol Scand. 1995;53(4): 259-263. doi: 10.3109/00016359509005983. | magnetic flux density not provided |
|  | Linder-Aronson S, Lindskog S. A morphometric study of bone surfaces and skin reactions after stimulation with static magnetic fields in rats. Am J Orthod Dentofacial Orthop. 1991;99(1): 44-48. doi: 10.1016/S0889-5406(05)81679-8. | magnetic flux density not provided |
|  | Lozano-Soto E, Soto-León V, Sabbarese S, Ruiz-Alvarez L, Sanchez-Del-Rio M, Aguilar J, et al. Transcranial static magnetic field stimulation (tSMS) of the visual cortex decreases experimental photophobia. Cephalalgia. 2018;38(8): 1493-1497. doi: 10.1177/0333102417736899. | magnetic flux density not provided |
|  | Luo R, Zhang Y, Xia L. Electrophysiological modeling study of ECG T-wave alternation caused by ultrahigh static magnetic fields. In: 2005 IEEE Engineering in Medicine and Biology 27th Annual Conference; 2005 Jan 17-18; Shanghai, China. 2005. p. 3012-3015. doi: 10.1109/IEMBS.2005.1617108. | other experimental study (modeling) |
|  | Makowski J, Oledzka-Slotwińska H, Gonet B. Effect of constant magnetic field on rat hepatocytes. Ann Med Sect Pol Acad Sci . 1976;21(1-2): 79-80. | > 1 mT |
|  | Man D, Man B, Plosker H. The influence of permanent magnetic field therapy on wound healing in suction lipectomy patients: a double-blind study. Plast Reconstr Surg. 1999;104(7): 2261-2266. | > 1 mT |
|  | Marhold S, Wiltschko W, Burda H. A Magnetic Polarity Compass for Direction Finding in a Subterranean Mammal. Naturwissenschaften. 1997;84(9): 421-423. doi: 10.1007/s001140050422. | (mechanisms of) magnetoreception |
|  | Martel GF, Andrews SC, Roseboom CG. Comparison of static and placebo magnets on resting forearm blood flow in young, healthy men. J Orthop Sports Phys Ther. 2002;32(10): 518-524. doi: 10.2519/jospt.2002.32.10.518. | > 1 mT |
|  | Martin FB, Bender A, Steuernagel G, Robinson RA, Revsbech R, Sorensen DK, et al. Epidemiologic study of Holstein dairy cow performance and reproduction near a high-voltage direct-current powerline. J Toxicol Environ Health. 1986;19(3): 303-324. doi: 10.1080/15287398609530930. | magnetic flux density not provided |
|  | Martínez-Bretón JL, Mendoza B, Miranda-Anaya M, Durán P, Flores-Chávez PL. Artificial reproduction of magnetic fields produced by a natural geomagnetic storm increases systolic blood pressure in rats. Int J Biometeorol. 2016;60(11): 1753-1760. doi: 10.1007/s00484-016-1164-5. | geomagnetic storm |
|  | Martini S, Begall S, Findeklee T, Schmitt M, Malkemper EP, Burda H. Dogs can be trained to find a bar magnet. PeerJ. 2018;6: e6117. doi: 10.7717/peerj.6117. | (mechanisms of) magnetoreception |
|  | Matsugi A, Okada Y. Cerebellar transcranial static magnetic field stimulation transiently reduces cerebellar brain inhibition. Funct Neurol. 2017;32(2): 77-82. doi: 10.11138/FNeur/2017.32.2.077. | > 1 mT |
|  | McCleary VL, Akers TK, Aasen GH. Low magnetic field effects on embryonic bone growth. Biomed Sci Instrum. 1991(27): 205-217. | other experimental study (technics) |
|  | McCleave JD, Power JH. Influence of weak electric and magnetic fields on turning behavior in elvers of the American eel *Anguilla rostrata*. Mar Biol. 1978;46(1): 29-34. | field deprivation/hypomagnetic field |
|  | McKay BE, Persinger MA. Complex magnetic fields enable static magnetic field cue use for rats in radial maze tasks. Int J Neurosci. 2005;115(5): 625-648. doi: 10.1080/00207450590523945. | (mechanisms of) magnetoreception |
|  | McLean MJ, Engstrom S, Holcomb RR, Sanchez D. A static magnetic field modulates severity of audiogenic seizures and anticonvulsant effects of phenytoin in DBA/2 mice. Epilepsy Res. 2003;55(1-2): 105-116. doi: 10.1016/s0920-1211(03)00109-8. | magnetic flux density of control group not provided |
|  | Mikesky AE, Hayden MW. Effect of static magnetic therapy on recovery from delayed onset muscle soreness. Phys Ther Sport. 2005;6(4): 188-194. doi: 10.1016/j.ptsp.2005.08.004. | > 1 mT |
|  | Mincheva T, Chifchiiski S, Genkov D. Cytomorphological and cytochemical studies of the reactive cells in the peritoneal cavity after experimental inflammation and exposure to a constant magnetic field (CMF). Folia Med (Plovdiv). 1984;26(1): 29-37. | > 1 mT |
|  | Mincheva T, Ishev V, Genkov D. Influence of a constant magnetic field on antibody formation in experimentally immunized white rats. Folia Med (Plovdiv). 1985;27(4): 44-47. | > 1 mT |
|  | Morris CE, Skalak TC. Chronic static magnetic field exposure alters microvessel enlargement resulting from surgical intervention. J Appl Physiol. 2007;103(2): 629-636. doi: 10.1152/japplphysiol.01133.2006. | > 1 mT |
|  | Muheim R, Edgar NM, Sloan KA, Phillips JB. Magnetic compass orientation in C57BL/6J mice. Learn Behav. 2006;34(4): 366-373. doi: 10.3758/bf03193201. | (mechanisms of) magnetoreception |
|  | Mühlbauer W. Der Einfluß magnetischer Felder auf die Wundheilung. Langenbecks Arch Chir. 1974;337(1): 637-642. doi: 10.1007/BF01278737. | > 1 mT, exposure condition unclear, not clear if peer-reviewed |
|  | Müller S, Hotz M. Human brainstem auditory evoked potentials (BAEP) before and after MR examinations. Magn Reson Med. 1990;16(3): 476-480. doi: 10.1002/mrm.1910160314. | > 1 mT |
|  | Murayama M. Orientation of sickled erythrocytes in a magnetic field. Nature. 1965;206(4982): 420-422. doi: 10.1038/206420a0. | other experimental study (in vitro), > 1 mT |
|  | Naito Y, Yamada S, Jinno Y, Arai K, Galli S, Ichikawa T, Jimbo R. Bone-Forming Effect of a Static Magnetic Field in Rabbit Femurs. Int J Periodontics Restorative Dent. 2019;39(2): 259-264. doi: 10.11607/prd.3220. | > 1 mT |
|  | Nakagawa K, Sasaki A, Nakazawa K. Accuracy in Pinch Force Control Can Be Altered by Static Magnetic Field Stimulation Over the Primary Motor Cortex. Neuromodulation. 2019;22(8): 871-876. doi: 10.1111/ner.12912. | > 1 mT |
|  | Nakagawa M. Detection of electrophysiological responses in rabbits affected by short-term exposure to static magnetic field. Nihon Eiseigaku Zasshi. 1984;38(6): 899-908. doi: 10.1265/jjh.38.899. | not English/German |
|  | Nakagawa M. Food consumption of mice in the static magnetic fields of moderate strength. Sangyo Igaku. 1980;22(4): 280-281. doi: 10.1539/joh1959.22.280. | > 1 mT |
|  | Nemec P, Altmann J, Marhold S, Burda H, Oelschläger HHA. Neuroanatomy of Magnetoreception: The Superior Colliculus Involved in Magnetic Orientation in a Mammal. Science. 2001;294(5541): 366-368. doi: 10.1126/science.1063351. | (mechanisms of) magnetoreception |
|  | Neurath PW. High gradient magnetic field inhibits embryonic development of frogs. Nature. 1968;219(5161): 1358-1359. doi: 10.1038/2191358a0. | > 1 mT |
|  | Newton KC, Kajiura SM. Magnetic field discrimination, learning, and memory in the yellow stingray (*Urobatis jamaicensis*). Anim Cogn. 2017;20(4): 603-614. doi: 10.1007/s10071-017-1084-8. | (mechanisms of) magnetoreception |
|  | Nikolskaya KA, Yeshchenko OV, Pratusevich V. The Opioid System and Magnetic Field Perception. Electro Magnetobiol. 1999;18(3): 277-290. doi: 10.3109/15368379909022584. | (mechanisms of) magnetoreception |
|  | Noda Y, Mori A, Liburdy RP, Packer L. Pulsed magnetic fields enhance nitric oxide synthase activity in rat cerebellum. Pathophysiology. 2000;7(2): 127-130. doi: 10.1016/s0928-4680(00)00039-0. | other experimental study (in vitro) |
|  | Nojima I, Koganemaru S, Fukuyama H, Mima T. Static magnetic field can transiently alter the human intracortical inhibitory system. Clin Neurophysiol. 2015;126(12): 2314-2319. doi: 10.1016/j.clinph.2015.01.030. | > 1 mT |
|  | Nojima I, Koganemaru S, Mima T. Combination of Static Magnetic Fields and Peripheral Nerve Stimulation Can Alter Focal Cortical Excitability. Front Hum Neurosci. 2016;10: 598. doi: 10.3389/fnhum.2016.00598. | > 1 mT |
|  | Okano H, Gmitrov J, Ohkubo C. Biphasic effects of static magnetic fields on cutaneous microcirculation in rabbits. Bioelectromagnetics. 1999;20(3): 161-171. doi: 10.1002/(SICI)1521-186X(1999)20:3<161::AID-BEM2>3.0.CO;2-O. | co-exposure, control group for SMF alone missing |
|  | Olcese J, Hurlbut E. Comparative studies on the retinal dopamine response to altered magnetic fields in rodents. Brain Res. 1989;498(1): 145-148. doi: 10.1016/0006-8993(89)90410-1. | (mechanisms of) magnetoreception |
|  | Olcese J, Reuss S, Stehle J, Steinlechner S, Vollrath L. Responses of the mammalian retina to experimental alteration of the ambient magnetic field. Brain Res. 1988;448(2): 325-330. doi: 10.1016/0006-8993(88)91271-1. | (mechanisms of) magnetoreception |
|  | Olcese J, Reuss S, Vollrath L(1985): Evidence for the involvement of the visual system in mediating magnetic field effects on pineal melatonin synthesis in the rat. Brain Res. 1985;333(2): 382-384. doi: 10.1016/0006-8993(85)91598-7. | (mechanisms of) magnetoreception |
|  | Olcese J, Reuss S. Magnetic field effects on pineal gland melatonin synthesis: comparative studies on albino and pigmented rodents. Brain Res. 1986;369(1-2): 365-368. doi: 10.1016/0006-8993(86)90552-4. | (mechanisms of) magnetoreception |
|  | Oliviero A, Carrasco-López MC, Campolo M, Perez-Borrego YA, Soto-León V, Gonzalez-Rosa JJ, et al. Safety Study of Transcranial Static Magnetic Field Stimulation(tSMS) of the Human Cortex. Brain Stimul. 2015;8(3): 481-485. doi: 10.1016/j.brs.2014.12.002. | > 1 mT |
|  | Oliviero A, Mordillo-Mateos L, Arias P, Panyavin I, Foffani G, Aguilar J. Transcranial static magnetic field stimulation of the human motor cortex. J Physiol. 2011;589(20): 4949-4958. doi: 10.1113/jphysiol.2011.211953. | > 1 mT |
|  | Orekhova NM, Akchurin RS, Belyaev AA, Smirnov MD, Ragimov SE, Orekhov AN. Local prevention of thrombosis in animal arteries by means of magnetic targeting of aspirin-loaded red cells. Thromb Res. 1990;57(4): 611-616. doi: 10.1016/0049-3848(90)90078-q. | other experimental study (in vitro) |
|  | Pan X, Xiao D, Zhang X, Huang Y, Lin B. Study of rotating permanent magnetic field to treat steroid-induced osteonecrosis of femoral head. Int Orthop. 2009;33(3): 617-623. doi: 10.1007/s00264-007-0506-7. | > 1 mT, no SMF |
|  | Papi F, Luschi P, Limonta P. Orientation-Disturbing Magnetic Treatment Affects the Pigeon Opioid System. J Exp Biol. 1992;166: 169-179. | (mechanisms of) magnetoreception |
|  | Pelyhe I, Mészáros I, Sárvári E. Effect of static magnetic field on the establishment of conditioned electrodefensive reflex in the rat. Acta Physiol Acad Sci Hung. 1973;43(2): 125-132. | magnetic flux density of control group not provided |
|  | Persson BR, Salford LG, Brun A, Eberhardt JL, Malmgren L. Increased permeability of the blood-brain barrier induced by magnetic and electromagnetic fields. Ann N Y Acad Sci. 1992; 649: 356-358. doi: 10.1111/j.1749-6632.1992.tb49629.x. | > 1 mT |
|  | Peterson HP, von Wangenheim KH, Feinendegen LE. Magnetic field exposure of marrow donor mice can increase the number of spleen colonies (CFU-S 7d) in marrow recipient mice. Radiat Environ Biophys. 1992;31(1): 31-38. doi: 10.1007/bf01211510. | > 1 mT |
|  | Phillips JB, Youmans PW, Muheim R, Sloan KA, Landler L, Painter MS, et al. Rapid learning of magnetic compass direction by C57BL/6 mice in a 4-armed 'plus' water maze. PLoS One. 2013;8(8): e73112. doi: 10.1371/journal.pone.0073112. | (mechanisms of) magnetoreception |
|  | Piruzyan LA, Rozenfel'd MA, Glezer VM, Lomonosov VA. Microcalorimetry of the processes of coagulation in normal conditions and after exposure to a constant magnetic field. Aerosp Med. 1969;40(10): 1140-1141. | > 1 mT |
|  | Pishchalnikov RY, Gurfinkel YI, Sarimov RM, Vasin AL, Sasonko ML, Matveeva TA, et al. Cardiovascular response as a marker of environmental stress caused by variations in geomagnetic field and local weather. Biomed Signal Process Control. 2019;51: 401-410. doi: 10.1016/j.bspc.2019.03.005. | geomagnetic storm |
|  | Politanski P, Rajkowska E, Pawlaczyk-Luszczynska M, Dudarewicz A, Wiktorek-Smagur A, Sliwinska-Kowalska M, et al. Static magnetic field affects oxidative stress in mouse cochlea. Int J Occup Med Environ Health. 2010;23(4): 377-384. doi: 10.2478/v10001-010-0041-4. | > 1 mT |
|  | Polyakov V, Trofimov A. Biorhythmological and clinico-functional features of arterial hypertension under geoecological conditions of the North. Alaska Med. 2007;49(2 Suppl): 120-126. | exposure condition unclear |
|  | Pope KW, McNally RJ. Nonspecific placebo effects explain the therapeutic benefit of magnets. The Scientific Review of Alternative Medicine. 2002;6(1): 13-16. | > 1 mT |
|  | Potenza L, Cucchiarini L, Piatti E, Angelini U, Dacha M. Effects of high static magnetic field exposure on different DNAs. Bioelectromagnetics. 2004;25(5): 352-355. doi: 10.1002/bem.10206. | other experimental study (in vitro) |
|  | Prato FS, Desjardins-Holmes D, Keenliside LD, Demoor JM, Robertson JA, Thomas AW. Magnetoreception in laboratory mice: sensitivity to extremely low-frequency fields exceeds 33 nT at 30 Hz. J R Soc Interface. 2013;10(81): 20121046. doi: 10.1098/rsif.2012.1046. | field deprivation/hypomagnetic field (for control group) |
|  | Puricelli E, Dutra NB, Ponzoni D. Histological evaluation of the influence of magnetic field application in autogenous bone grafts in rats. Head Face Med. 2009;5: 1. doi: 10.1186/1746-160X-5-1. | > 1 mT |
|  | Puricelli E, Ulbrich LM, Ponzoni D, Filho JJ. Histological analysis of the effects of a static magnetic field on bone healing process in rat femurs. Head Face Med. 2006;2: 43. doi: 10.1186/1746-160X-2-43. | > 1 mT |
|  | Raybourn MS. The effects of direct-current magnetic fields on turtle retinas in vitro. Science. 1983;220(4598): 715-717. doi: 10.1126/science.6682247. | > 1 mT |
|  | Reiter RJ, Tan DX, Poeggeler B, Kavet R. Inconsistent suppression of nocturnal pineal melatonin synthesis and serum melatonin levels in rats exposed to pulsed DC magnetic fields. Bioelectromagnetics. 1998;19(5): 318-329. doi: 10.1002/(sici)1521-186x(1998)19:5<318::aid-bem6>3.0.co;2-4. | no SMF |
|  | Reno VR, Nutini LG. Effect of magnetic fields on tissue respiration. Nature. 1963;198(4876): 204-205. doi: 10.1038/198204b0. | > 1 mT |
|  | Reuss S, Olcese J. Magnetic field effects on the rat pineal gland: role of retinal activation by light. Neurosci Lett. 1986;64(1): 97-101. doi: 10.1016/0304-3940(86)90670-1. | (mechanisms of) magnetoreception |
|  | Reuss S, Semm P, Vollrath L. Different types of magnetically sensitive cells in the rat pineal gland. Neurosci Lett. 1983;40(1): 23-26. doi: 10.1016/0304-3940(83)90086-1. | (mechanisms of) magnetoreception |
|  | Roberts DC, Marcelli V, Gillen JS, Carey JP, Della Santina CC, Zee DS. MRI magnetic field stimulates rotational sensors of the brain. Curr Biol. 2011;21(19): 1635-1640. doi: 10.1016/j.cub.2011.08.029. | MRI |
|  | Rudolph K, Wirz-Justice A, Kräuchi K, Feer H. Static magnetic fields decrease nocturnal pineal cAMP in the rat. Brain Res. 1988;446(1): 159-160. doi: 10.1016/0006-8993(88)91307-8. | (mechanisms of) magnetoreception |
|  | Russo A, Bianchi M, Sartori M, Parrilli A, Panseri S, Ortolani A, et al. Magnetic forces and magnetized biomaterials provide dynamic flux information during bone regeneration. J Mater Sci Mater Med. 2016;27(3): 51. doi: 10.1007/s10856-015-5659-0. | > 1 mT, co-exposure (magnetic scaffold) |
|  | Saeedi Goraghani M, Ahmadi-Zeidabadi M, Bakhshaei S, Shabani M, Ghotbi Ravandi S, Rezaei Zarchi S, et al. Behavioral consequences of simultaneous postnatal exposure to MK-801 and static magnetic field in male Wistar rats. Neurosci Lett. 2019;701: 77-83. doi: 10.1016/j.neulet.2019.02.026. | > 1 mT |
|  | Sakata M, Yamamoto Y, Imamura N, Nakata S, Nakasima A. The effects of a static magnetic field on orthodontic tooth movement. J Orthod. 2008;35(4): 249-254. doi: 10.1179/14653120722752. | > 1 mT |
|  | Salem A, Hafedh A, Rached A, Mohsen S, Khemais BR. Zinc prevents hematological and biochemical alterations induced by static magnetic field in rats. Pharmacol Rep. 2005;57(5): 616-622. | > 1 mT |
|  | Salim EI, Omar KM, Abou-Hattab HA, Abou-Zaid FA(2008): Pituitary toxicity but lack of rat colon carcinogenicity of a DC-magnetic field in a medium-term bioassay. Asian Pac J Cancer Prev. 2008;9(1): 131-140. | co-exposure |
|  | Salvatore JR, Harrington J, Kummet T. Phase I clinical study of a static magnetic field combined with anti-neoplastic chemotherapy in the treatment of human malignancy: Initial safety and toxicity data. Bioelectromagnetics. 2003;24(7): 524-527. doi: 10.1002/bem.10149. | > 1 mT |
|  | Sándor K, Helyes Z, Gyires K, Szolcsányi J, László J. Static magnetic field-induced anti-nociceptive effect and the involvement of capsaicin-sensitive sensory nerves in this mechanism. Life Sci. 2007;81(2): 97-102. doi: 10.1016/j.lfs.2007.04.029. | > 1 mT |
|  | Saygili G, Aydinlik E, Ercan MT, Naldöken S, Ulutuncel N. Investigation of the effect of magnetic retention systems used in prostheses on buccal mucosal blood flow. Int J Prosthodont. 1992;5(4): 326-332. | > 1 mT |
|  | Schaap K, Portengen L, Kromhout H. Exposure to MRI-related magnetic fields and vertigo in MRI workers. Occup Environ Med. 2016;73(3): 161-166. doi: 10.1136/oemed-2015-103019. | MRI |
|  | Schlegel PA. Magnetic and other non-visual orientation mechanisms in some cave and surface urodeles. J Ethol. 2008;26(3): 347-359. doi: 10.1007/s10164-007-0071-y. | (mechanisms of) magnetoreception |
|  | Schwartz JL. Influence of a constant magnetic field on nervous tissues: II. Voltage-clamp studies. IEEE Trans Biomed Eng. 1979;26(4): 238-243. doi: 10.1109/TBME.1978.326353. | other experimental study (invertebrate), > 1 mT |
|  | Schwartz M, Steidle G, Martirosian P, Ramos-Murguialday A, Preißl H, Stemmer A, et al. Spontaneous mechanical and electrical activities of human calf musculature at rest assessed by repetitive single-shot diffusion-weighted MRI and simultaneous surface electromyography. Magn Reson Med. 2018;79(5): 2784-2794. doi: 10.1002/mrm.26921. | MRI |
|  | Segal NA, Huston J, Fuchs H, Holcomb R, McLean MJ. Efficacy of a static magnetic device against knee pain associated with inflammatory arthritis. J Clin Rheumatol. 1999;5(5): 302-304. | > 1 mT |
|  | Segal NA, Toda Y, Huston J, Saeki Y, Shimizu M, Fuchs H, et al. Two configurations of static magnetic fields for treating rheumatoid arthritis of the knee: a double-blind clinical trial. Arch Phys Med Rehabil. 2001;82(10): 1453-1460. doi: 10.1053/apmr.2001.24309. | > 1 mT |
|  | Semm P, Beason RC. Responses to small magnetic variations by the trigeminal system of the bobolink. Brain Res Bull. 1990;25(5): 735-740. doi: 10.1016/0361-9230(90)90051-Z. | no SMF |
|  | Semm P, Demaine C. Neurophysiological properties of magnetic cells in the pigeon's visual system. J Comp Physiol A. 1986;159(5): 619-625. doi: 10.1007/bf00612035. | (mechanisms of) magnetoreception |
|  | Semm P, Demaine C. Neurophysiological properties of magnetic cells in the pigeon's visual system. J Comp Physiol A. 1986;159(5): 619-625. doi: 10.1007/BF00612035. | (mechanisms of) magnetoreception |
|  | Semm P, Nohr D, Demaine C, Wiltschko W. Neural basis of the magnetic compass: interactions of visual, magnetic and vestibular inputs in the pigeon's brain. J Comp Physiol A. 1984;155(3): 283-288. doi: 10.1007/BF00610581. | (mechanisms of) magnetoreception |
|  | Semm P, Schneider T, Vollrath L. Effects of an earth-strength magnetic field on electrical activity of pineal cells. Nature. 1980;288(5791): 607-608. doi: 10.1038/288607a0. | magnetic flux density of control group not provided |
|  | Sheffield A, Ahn S, Alagapan S, Fröhlich F. Modulating neural oscillations by transcranial static magnetic field stimulation of the dorsolateral prefrontal cortex: A crossover, double-blind, sham-controlled pilot study. Eur J Neurosci. 2019;49(2): 250-262. doi: 10.1111/ejn.14232. | > 1 mT |
|  | Shieh YY, Tsai FY. Static magnetotherapy for the treatment of insomnia. Int J Electron Healthc. 2008;4(3-4): 339-349. doi: 10.1504/IJEH.2008.02267. | > 1 mT |
|  | Siadat H, Bassir SH, Alikhasi M, Shayesteh YS, Khojasteh A, Monzavi A. Effect of Static Magnetic Fields on the Osseointegration of Immediately Placed Implants: A Randomized Controlled Clinical Trial. Implant Dent. 2012;21(6): 491-495. doi: 10.1097/ID.0b013e31826dcc2f. | > 1 mT |
|  | Silbert BI, Pevcic DD, Patterson HI, Windnagel KA, Thickbroom GW. Inverse correlation between resting motor threshold and corticomotor excitability after static magnetic stimulation of human motor cortex. Brain Stimul. 2013;6(5): 817-820. doi: 10.1016/j.brs.2013.03.007. | > 1 mT |
|  | Simoncini L, Giuriati L, Giannini S. Clinical evaluation of the effective use of magnetic fields in podology. Chir Organi Mov. 2001;86(3): 243-247. | > 1 mT |
|  | Singh P, YashRoy RC, Hoque M. Augmented bone-matrix formation and osteogenesis under magnetic field stimulation in vivo XRD, TEM and SEM investigations. Indian J Biochem Biophys. 2006;43(3): 167-172. | exposure condition unclear |
|  | Song BW, Hong H, Jung YJ, Lee JH, Kim BS, Lee HB. Combination Therapy Comprising a Static Magnetic Field with Contractility Improves Skin Wounds. Tissue Eng Part A. 2018;24(17-18): 1354-1363. doi: 10.1089/ten.TEA.2017.0470. | > 1 mT |
|  | Sorahan T. Cancer incidence in UK electricity generation and transmission workers, 1973-2008. Occup Med. 2012;62(7): 496-505. doi: 10.1093/occmed/kqs152. | magnetic flux density not provided |
|  | Sperber D, Oldenbourg R, Dransfeld K. Magnetic field induced temperature change in mice. Naturwissenschaften. 1984;71(2): 100-101. doi: 10.1007/bf01156362. | > 1 mT |
|  | Stefanov VE, Shchegolev BF, Kriyachko OV, Kuzmenko NV, Surma SV, Spivak IM. Model study of biological effects of weak static magnetic fields at the organismic and subcellular levels. Dokl Biol Sci. 2015;461(1): 116-119. doi: 10.1134/S0012496615020118. | field deprivation/hypomagnetic field |
|  | Stehle J, Reuss S, Schröder H, Henschel M, Vollrath L. Magnetic field effects on pineal N-acetyltransferase activity and melatonin content in the gerbil -- role of pigmentation and sex. Physiol Behav. 1988;44(1): 91-94. doi: 10.1016/0031-9384(88)90350-2. | (mechanisms of) magnetoreception |
|  | Stern S, Laties VG, Nguyen QA, Cox C. Exposure to combined static and 60 Hz magnetic fields: failure to replicate a reported behavioral effect. Bioelectromagnetics. 1996;17(4): 279-292. doi: 10.1002/(SICI)1521-186X(1996)17:4<279::AID-BEM4>3.0.CO;2-0. | field deprivation/hypomagnetic field, co-exposure |
|  | Steyn PF, Ramey DW, Kirschvink J, Uhrig J. Effect of a static magnetic field on blood flow to the metacarpus in horses. J Am Vet Med Assoc. 2000;217(6): 874-877. doi: 10.2460/javma.2000.217.874. | > 1 mT |
|  | Stojan L, Sperber D, Dransfeld K. Influence of high steady magnetic fields on the electrical activity of the electric fish Apteronotus. Z Naturforsch C. 1990;45(3-4): 303-305. | > 1 mT |
|  | Sun W, He Y, Leung SW, Kong YC. In Vivo Analysis of Embryo Development and Behavioral Response of Medaka Fish under Static Magnetic Field Exposures. Int J Environ Res Public Health. 2019;16(5): e844. doi: 10.3390/ijerph16050844. | > 1 mT |
|  | Suomi R, Koceja DM. Effect of magnetic insoles on postural sway measures in men and women during a static balance test. Percept Mot Skills. 2001;92(2): 469-476. doi: 10.2466/pms.2001.92.2.469. | > 1 mT |
|  | Swanson J, Kheifets L. Could the geomagnetic field be an effect modifier for studies of power-frequency magnetic fields and childhood leukaemia? J Radiol Prot. 2012;32(4): 413-418. doi: 10.1088/0952-4746/32/4/413. | co-exposure |
|  | Sweeney KB, Merrick MA, Ingersoll CD, Swez JA. Therapeutic Magnets Do Not Affect Tissue Temperatures. J Athl Train. 2001;36(1): 27-31. | > 1 mT |
|  | Szor JK, Holewinski P. Lessons learned in research: an attempt to study the effects of magnetic therapy. Ostomy Wound Manage. 2002;48(2): 24-29. | > 1 mT |
|  | Takeshige C, Sato M. Comparisons of pain relief mechanisms between needling to the muscle, static magnetic field, external qigong and needling to the acupuncture point. Acupunct Electrother Res. 1996;21(2): 119-131. doi: 10.3727/036012996816356924. | > 1 mT |
|  | Tamaki T, Yoshioka T, Nakano S. Effect of magnetic field on the contractility and glycogen content in neuromuscular preparation. Tokai J Exp Clin Med. 1987;12(1): 55-59. | other experimental study (in vitro) |
|  | Taniguchi N, Kanai S, Kawamoto M, Endo H, Higashino H. Study on Application of Static Magnetic Field for Adjuvant Arthritis Rats. Evid Based Complement Alternat Med. 2004;1(2): 187-191. doi: 10.1093/ecam/neh024. | > 1 mT |
|  | Taniguchi N, Kanai S. Efficacy of Static Magnetic Field for Locomotor Activity of Experimental Osteopenia. Evid Based Complement Alternat Med. 2007;4(1): 99-105. doi: 10.1093/ecam/nel067. | > 1 mT |
|  | Tanski A, Formicki K, Korzelecka-Orkisz A, Winnicki A. Spatial Orientation Of Fish Embryos In Magnetic Field. Electronic Journal of Ichthyology. 2005;.(1): 21-34. | (mechanisms of) magnetoreception |
|  | Tenforde TS. Mechanisms for Biological Effects of Magnetic Fields. In: Grandolfo M., Michaelson S.M., Rindi A, editors. Biological Effects and Dosimetry of Static and ELF Electromagnetic Fields. Ettore Majorana International Science Series. Springer: Boston, MA; 1985. p. 71-92. doi: 10.1007/978-1-4613-2099-9_5. | review, editorial, comment |
|  | Thomas JR, Schrot J, Liboff AR. Low-intensity magnetic fields alter operant behavior in rats. Bioelectromagnetics. 1986;7(4): 349-357. doi: 10.1002/bem.2250070402. | field deprivation/hypomagnetic field |
|  | Tian L, Zhang B, Zhang J, Zhang T, Cai Y, Qin H, et al. A magnetic compass guides the direction of foraging in a bat. J Comp Physiol A Neuroethol Sens Neural Behav Physiol. 2019;205(4): 619-627. doi: 10.1007/s00359-019-01353-1. | (mechanisms of) magnetoreception |
|  | Tis LL, Trinkhaus MD, Higbie EJ, Johnson BF, McCarty FA. Effects of magnets on concentric and eccentric isokinetic force production of the quadriceps group. Isokinet Exerc Sci. 2000;8(4): 217-221. doi: 10.3233/IES-2000-0055. | > 1 mT |
|  | Tofani S, Barone D, Berardelli M, Berno E, Cintorino M, Foglia L, et al. Static and ELF magnetic fields enhance the in vivo anti-tumor efficacy of *cis*-platin against lewis lung carcinoma, but not of cyclophosphamide against B16 melanotic melanoma. Pharmacol Res. 2003;48(1): 83-90. doi: 10.1016/S1043-6618(03)00062-8. | co-exposure |
|  | Togawa T, Okai O, Oshima M. Observation of blood flow E.M.F. in externally applied strong magnetic field by surface electrodes. Med Biol Eng. 1967;5(2): 169-170. doi: 10.1007/bf02474505. | > 1 mT |
|  | Vaena MLHT, Sinnecker JP, Vargas TJS, Serra-Guimarães F, Marques RG. Magnetic transcutaneous fixation: an experimental study in pigs. J Surg Res. 2017;220: 139-146. doi: 10.1016/j.jss.2017.06.081. | > 1 mT |
|  | Valles JM, Wasserman SR, Schweidenback C, Edwardson J, Denegre JM, Mowry KL. Processes that occur before second cleavage determine third cleavage orientation in Xenopus. Exp Cell Res. 2002;274(1): 112-118. doi: 10.1006/excr.2001.5456. | > 1 mT |
|  | Vargas JP, Siegel JJ, Bingman VP. The effects of a changing ambient magnetic field on single-unit activity in the homing pigeon hippocampus. Brain Res Bull. 2006;70(2): 158-164. doi: 10.1016/j.brainresbull.2006.03.018. | (mechanisms of) magnetoreception |
|  | Vesselinova L. Biosomatic Effects of the Electromagnetic Fields on View of the Physiotherapy Personnel Health. Electromagn Biol Med. 2013;32(2): 192-199. doi: 10.3109/15368378.2013.776429. | magnetic flux density not provided |
|  | Vesselinova L. Body mass index as a risk prediction and prevention factor for professional mixed low-intensity EMF burden. Electromagn Biol Med. 2015;34(3): 238-243. doi: 10.3109/15368378.2015.1076449. | magnetic flux density not provided |
|  | von Klitzing L, Gerhard H, Benthin U, Jörg J. Statische NMR-Magnetfelder verändern die somatosensibel evozierten Potentiale beim Menschen. EEG EMG Z Elektroenzephalogr Elektromyogr Verwandte Geb. 1987;18(1): 43-46. doi: 10.1055/s-2008-1060896. | MRI |
|  | von Klitzing L. A new encephalomagnetic effect in human brain generated by static magnetic fields. Brain Res. 1991;540(1-2): 295-296. doi: 10.1016/0006-8993(91)90522-w. | > 1 mT |
|  | von Klitzing L. Einfluß statischer Magnetfelder auf die Biosignalverarbeitung beim Menschen. Biomed Tech(Berl). 1990;35 Suppl 2: 17-19. | exposure condition unclear |
|  | von Klitzing L. Static magnetic fields increase the power intensity of EEG of man. Brain Res. 1989;483(1): 201-203. doi: 10.1016/0006-8993(89)90056-5. | > 1 mT |
|  | von Klitzing L. Werden periodisch evozierte Potentiale beim Menschen in statischen Magnetfeldern als Zeitfunktion gespeichert? Roentgenpraxis. 1988;41(5): 162-165. | > 1 mT |
|  | Vorobyov VV, Sosunov EA, Kukushkin NI, Lednev VV. Weak combined magnetic field affects basic and morphine-induced rat's EEG. Brain Res. 1998;781(1-2): 182-187. doi: 10.1016/s0006-8993(97)01228-6. | co-exposure |
|  | Walker MM, Diebel CE, Haugh CV, Pankhurst PM, Montgomery JC, Green CR. Structure and function of the vertebrate magnetic sense. Nature. 1997;390(6658): 371-376. doi: 10.1038/37057. | (mechanisms of) magnetoreception |
|  | Wang CX, Hilburn IA, Wu DA, Mizuhara Y, Cousté CP, Abrahams JNH, et al. Transduction of the Geomagnetic Field as Evidenced from alpha-Band Activity in the Human Brain. eNeuro. 2019;6(2): pii: ENEURO.0483-18.2019. doi: 10.1523/ENEURO.0483-18.2019. | (mechanisms of) magnetoreception |
|  | Wang Y, Pan Y, Parsons S, Walker M, Zhang S. Bats respond to polarity of a magnetic field. Proc R Soc B. 2007;274(1627): 2901-2905. doi: 10.1098/rspb.2007.0904. | (mechanisms of) magnetoreception |
|  | Ward B, Zee D. Dizziness and Vertigo during MRI. N Engl J Med. 2016;375(21): e44. doi: 10.1056/NEJMicm1514075. | MRI |
|  | Welker HA, Semm P, Willig RP, Commentz JC, Wiltschko W, Vollrath L. Effects of an artificial magnetic field on serotonin N-acetyltransferase activity and melatonin content of the rat pineal gland. Exp Brain Res. 1983;50(2-3): 426-432. doi: 10.1007/BF00239209. | (mechanisms of) magnetoreception |
|  | Wever R. The effects of electric fields on circadian rhythmicity in men. Life Sci Space Res. 1970;8: 177-187. | review, editorial, comment |
|  | Williams AE, Croft J, Napp V, Corrigan N, Brown JM, Hulme C, et al. SaFaRI: sacral nerve stimulation versus the FENIX magnetic sphincter augmentation for adult faecal incontinence: a randomised investigation. Int J Colorectal Dis. 2016;31(2): 465-472. doi: 10.1007/s00384-015-2492-3. | not EMF/health-related |
|  | Wilson BW, Wright CW, Morris JE, Buschbom RL, Brown DP, Miller DL, et al. Evidence for an effect of ELF electromagnetic fields on human pineal gland function. J Pineal Res. 1990;9(4): 259-269. doi: 10.1111/j.1600-079x.1990.tb00901.x. | magnetic flux density of control group not provided |
|  | Wiltschko R, Wiltschko W. Evidence for the use of magnetic outward-journey information in homing pigeons. Naturwissenschaften. 1978;65(2): 112-113. doi: 10.1007/BF00440557. | (mechanisms of) magnetoreception |
|  | Wiltschko W. Über den Einfluß statischer Magnetfelder auf die Zugorientierung der Rotkehlchen (*Erithacus rubecula*). Z Tierpsychol. 1968;25(5): 537-558. doi: 10.1111/j.1439-0310.1968.tb00028.x. | (mechanisms of) magnetoreception |
|  | Winemiller MH, Billow RG, Laskowski ER, Harmsen WS. Effect of magnetic vs sham-magnetic insoles on nonspecific foot pain in the workplace: a randomized, double-blind, placebo-controlled trial. Mayo Clin Proc. 2005;80(9): 1138-1145. doi: 10.4065/80.9.1138. | > 1 mT |
|  | Winemiller MH, Billow RG, Laskowski ER, Harmsen WS. Effect of magnetic vs sham-magnetic insoles on plantar heel pain: a randomized controlled trial. JAMA. 2003;290(11): 1474-1478. doi: 10.1001/jama.290.11.1474. | > 1 mT |
|  | Wolsko PM, Eisenberg DM, Simon LS, Davis RB, Walleczek J, Mayo-Smith M et al. Double-blind placebo-controlled trial of static magnets for the treatment of osteoarthritis of the knee: results of a pilot study. Altern Ther Health Med. 2004;10(2): 36-43. | > 1 mT |
|  | Wordsworth OJ(1974): Comparative long-term effects of liver damage in the rat after (a) localized X-irradiation and (b) localized X-irradiation in the presence of a strong homogeneous magnetic field. Radiat Res. 1974;57(3): 442-450. doi: 10.2307/3573891. | > 1 mT |
|  | Wu LQ, Dickman JD. Magnetoreception in an avian brain in part mediated by inner ear lagena. Curr Biol. 2011;21(5): 418-423. doi: 10.1016/j.cub.2011.01.058. | (mechanisms of) magnetoreception |
|  | Xu S, Okano H, Nakajima M, Hatano N, Tomita N, Ikada Y(2013): Static magnetic field effects on impaired peripheral vasomotion in conscious rats. Evid Based Complement Alternat Med. 2013: pii:746968-1-746968-6. doi: 10.1155/2013/746968. | > 1 mT |
|  | Xu S, Tomita N, Ikeuchi K, Ikada Y. Recovery of small-sized blood vessels in ischemic bone under static magnetic field. Evid Based Complement Alternat Med. 2007;4(1): 59-63. doi: 10.1093/ecam/nel055. | > 1 mT |
|  | Xu S, Tomita N, Ohata R, Yan Q, Ikada Y. Static magnetic field effects on bone formation of rats with an ischemic bone model. Biomed Mater Eng. 2001;11(3): 257-263. | > 1 mT |
|  | Yaga K, Reiter RJ, Manchester LC, Nieves H, Sun JH, Chen LD. Pineal sensitivity to pulsed static magnetic fields changes during the photoperiod. Brain Res Bull. 1993;30(1-2): 153-156. doi: 10.1016/0361-9230(93)90052-d. | no SMF |
|  | Yan QC, Tomita N, Ikada Y. Effects of static magnetic field on bone formation of rat femurs. Med Eng Phys. 1998;20(6): 397-402. doi: 10.1016/s1350-4533(98)00051-4. | > 1 mT |
|  | Yano A, Ogura M, Sato A, Sakaki Y, Ban M, Nagasawa K. Development of ultrasonic telemetry technique for investigating the magnetic sense of salmonids. Fish Sci. 1996;62(5): 698-704. doi: 10.2331/fishsci.62.698. | (mechanisms of) magnetoreception |
|  | Yano A, Ogura M, Sato A, Sakaki Y, Shimizu Y, Baba N, et al. Effect of modified magnetic field on the ocean migration of maturing chum salmon, *Oncorhynchus keta*. Mar Biol. 1997;129(3): 523-530. doi: 10.1007/s002270050193. | (mechanisms of) magnetoreception |
|  | Yokoi I, Kabuto H, Nanba Y, Yamamoto N, Ogawa N, Mori A. Alternate magnetic fields potentiate monoamine oxidase activity in the brain. Pathophysiology. 2000;7(2): 121-125. doi: 10.1016/S0928-4680(00)00038-9. | other experimental study (in vitro) |
|  | Zagalskaya EO, Gnyubkina VP, Maksimovich AA. Morphological Characteristics of the Retinomotor Response in Salmon Trout (*Oncorhynchus Masou*) Fry in a Magnetic Field and Red Light. Neurosci Behav Physiol. 2005;35(9): 903-907. doi: 10.1007/s11055-005-0143-9. | other experimental study (in vitro), magnetic flux density not provided |
|  | Zhadin MN, Deryugina ON, Pisachenko TM. Influence of combined DC and AC magnetic fields on rat behavior. Bioelectromagnetics.1999;20(6): 378-386. doi: 10.1002/(sici)1521-186x(199909)20:6<378::aid-bem7>3.0.co;2-0. | magnetic flux density of control group not provided |
|  | Zhang J, Meng X, Ding C, Shang P. Effects of static magnetic fields on bone microstructure and mechanical properties in mice. Electromagn Biol Med. 2018;37(2): 76-83. doi: 10.1080/15368378.2018.1458626. | > 1 mT |
|  | Zhang X, Luo Z, He X, Han Q, Zhang W. Molecular mechanism of effect of rotating constant magnetic field on organisms. Sci China C Life Sci. 2001;44(5): 554-560. doi: 10.1007/BF02882398. | other experimental study (in vitro) |
|  | Zhernovoi AI, Skorik VI, Chirukhin VA, Sharshina LM. Effect of stationary magnetic field on in vivo oxygen binding by blood. Bull Exp Biol Med.2001;131(2): 121-123. doi: 10.1023/a:1017523407969. | > 1 mT |
|  | Zhu Y, Wang S, Long H, Zhu J, Jian F, Ye N, et al. Effect of static magnetic field on pain level and expression of P2X3 receptors in the trigeminal ganglion in mice following experimental tooth movement. Bioelectromagnetics. 2017;38(1): 22-30. doi: 10.1002/bem.22009. | > 1 mT |
