# Supplementary material for: Biological and health-related effects of weak static magnetic fields (≤ 1 mT) in humans and vertebrates: A systematic review
Source: PLoS One. 2020 Jun 9;15(6):e0230038. doi: 10.1371/journal.pone.0230038 (PMC7282627; doi:10.1371/journal.pone.0230038)
Supplement: S1 Link — (DOCX) [file pone.0230038.s005.docx]

**S1 Link. Search string for systematic literature search in the EMF-Portal**

<https://www.emf-portal.org/de/article/search/results?keywords=%22static+magnetic+field%22&logicalOperator=0&authors=&journals=&years=&topics%5B%5D=0&topics%5B%5D=1&topics%5B%5D=2&topics%5B%5D=3&topics%5B%5D=4&topics%5B%5D=5&topics%5B%5D=6&topics%5B%5D=7&topics%5B%5D=8&topics%5B%5D=9&frequencyRanges%5B%5D=0&frequencyRanges%5B%5D=1&frequencyRanges%5B%5D=2&frequencyRanges%5B%5D=3&frequencyRanges%5B%5D=4&timeSpan=0>

last accessed in march 2018
