# Supplementary material for: Biological and health-related effects of weak static magnetic fields (≤ 1 mT) in humans and vertebrates: A systematic review
Source: PLoS One. 2020 Jun 9;15(6):e0230038. doi: 10.1371/journal.pone.0230038 (PMC7282627; doi:10.1371/journal.pone.0230038)
Supplement: S2 Link — (DOCX) [file pone.0230038.s006.docx]

**Literature search**

The electronic database searches can be repeated using the following links and search strings:

**PubMed**

<https://www.ncbi.nlm.nih.gov/pubmed/?term=%22static+magnetic+field%22+OR+%22static+magnetic+fields%22+OR+%22DC+magnetic+field%22+OR+%22DC+magnetic+fields%22+OR+%22constant+magnetic+field%22+OR+%22constant+magnetic+fields%22+OR+%22steady+magnetic+field%22+OR+%22steady+magnetic+fields%22+OR+%22stationary+magnetic+field%22+OR+%22stationary+magnetic+fields%22+OR+%22magnetostatic+field%22+OR+%22magnetostatic+fields%22+OR+%22high-voltage+direct+current%22+OR+HVDC>

"static magnetic field" OR "static magnetic fields" OR "DC magnetic field" OR "DC magnetic fields" OR "constant magnetic field" OR "constant magnetic fields" OR "steady magnetic field" OR "steady magnetic fields" OR "stationary magnetic field" OR "stationary magnetic fields" OR "magnetostatic field" OR "magnetostatic fields" OR "high-voltage direct current" OR HVDC

**EMF-Portal**

<https://www.emf-portal.org/en/article/search/results?keywords=%22static+magnetic+field%22+%22DC+magnetic+field%22+%22steady+magnetic+field%22+%22stationary+magnetic+field%22+%22magnetostatic+field%22+HG%C3%9C+%22high-voltage+direct+current%22&logicalOperator=1&authors=&authorMatchingMode=0&journals=&journalMatchingMode=0&years=&topics%5B%5D=0&topics%5B%5D=1&topics%5B%5D=2&topics%5B%5D=3&topics%5B%5D=4&topics%5B%5D=5&topics%5B%5D=6&topics%5B%5D=7&topics%5B%5D=8&topics%5B%5D=9&frequencyRanges%5B%5D=0&frequencyRanges%5B%5D=1&frequencyRanges%5B%5D=2&frequencyRanges%5B%5D=3&frequencyRanges%5B%5D=4&frequencyRanges%5B%5D=5&timeSpan=0>

"static magnetic field" "DC magnetic field" "steady magnetic field" "stationary magnetic field" "magnetostatic field" HGÜ "high-voltage direct current"
